# Exogenously delivered heat shock protein 70 displaces its endogenous analogue and sensitizes cancer cells to lymphocytes-mediated cytotoxicity

**DOI:** 10.18632/oncotarget.1820

**Published:** 2014-03-22

**Authors:** Maxim A. Shevtsov, Elena Y. Komarova, Darya A. Meshalkina, Natalia V. Bychkova, Nikolai D. Aksenov, Sergey V. Abkin, Boris A. Margulis, Irina V. Guzhova

**Affiliations:** ^1^ Institute of Cytology of Russian Academy of Sciences, St. Petersburg, Russia; ^2^ Laboratory of Clinical Immunology, EMERCOM of Russia, St.Petersburg, Russia

**Keywords:** heat shock protein 70, intra-, extracellular transport, cytotoxic lymphocytes, cancer cell

## Abstract

Hsp70 chaperone is known to stimulate anti-tumour immunity in a variety of cancer models. Here we demonstrated that the addition of purified recombinant Hsp70 to the culture medium facilitated cancer cell cytolysis by lymphocytes. Importantly, exogenous Hsp70 triggered secretion of the intracellular Hsp70 to a cell surface and extracellular milieu, which played a role in cytolysis because down-regulation of the endogenous Hsp70 reduced both its presence at the cell surface and the lymphocyte-mediated cytolysis. Inhibitors that target both the ATPase and the peptide-binding domains of Hsp70 molecule potently decreased its anti-tumor effect. Using a variety of cell transport markers and inhibitors, we showed that the exchange of exogenous and intracellular Hsp70 is supported by classical and non-classical transport pathways, with a particular role of lipid rafts in the chaperone's intracellular transport. In conclusion, exogenous Hsp70 can eject endogenous Hsp70, thus exerting anticancer activity.

## INTRODUCTION

Heat shock proteins, particularly Hsp70, play a dual role in cancer cells: the elevation of their content enhances cell protection to a variety of cytotoxic factors, while cells over-expressing Hsp70 have been shown to transport the chaperone to the surface which leads to their sensitization to specific and non-specific immune responses [1]. At an earlier stage of the chaperone-regulated immunomodulatory process, Hsp70 induced by a certain factor – heat stress for instance – may expose on the outer membrane of a cancer cell its 14-amino acid sequence (TKD peptide) found to be a target for pre-activated NK cells [2]. Stimulation of tumour cells to apoptosis also leads to exposition of Hsp70 on cell surface [3] and recognition of surface Hsp70 by splenic cytotoxic cells [4]. Similarly, the specific response of CD4- and/or CD8-positive cells to tumour can be triggered by Hsp70 released from dying or alive cancer cells [5,6]. On the other hand the mobilization of the specific immune response is associated with the adjuvant activity of the chaperone able to carry tumour or viral antigens and present these to dendrytic cells followed by the initiation of cytokine production, up-regulation of cytotoxic activity and infiltration of a tumour with CD4+ and CD8+-positive lymphocytes [7]. Innate immunity can also be triggered by the exogenous Hsp70 (exo-Hsp70), as proven in experiments where pure recombinant chaperone was shown to activate NF-kappaB factor system through TLR2/TLR4 [8,9]. Thus to elicit its immunomodulatory potential, Hsp70 should be present outside a cancer cell, suggesting that the mechanism of the chaperone's reaction with the cell is of great importance [10]. The effects of exo-Hsp70 on a cell were shown to depend on the cell type as well as on the nature or concentration of the protein. It was found that exogenously occurring Hsp70 can enter a neural cell and protect it from the deleterious effect of hyperthermia or apoptosis inducer, staurosporine [11], or inhibit the growth of aggregates of mutant huntingtin with abnormally long polyglutamine tracts [12]. On the contrary, Hsp70 was able to induce apoptosis in PC-12 cells by interacting with phosphatidylserine moiety of plasma membrane [13]. Additionally, some effects of exogenous Hsp70 can be related to its recognition by Lox-1 and SREC scavenger receptors or TLR2/TLR4 innate immune receptors [14].

The multiple activities of Hsp70 introduced into the culture of cancer cells are of practical interest because a few anti-tumour vaccines have been constructed to date based on the exogenously delivered chaperone. One of the vaccines constitutes a specific line of murine ovarian cancer cells constantly secreting Hsp70 [15]. Wang with co-authors proposed an AdSurp-Hsp70 viral therapy system used to regulate the selective lysis of tumor cells and Hsp70-mediated elevation of immune response [16]. Another vaccine construct is based on the fusion of Hsp70 with the Herpes virus VP22 peptide (aa 268–301) that facilitates intracellular transport [17]. The system developed by Ito and others includes intra-tumourally injected pure Hsp70 and heating magnetic particles; this vaccine can efficiently destroy B16 mouse melanoma in a therapeutic modality [18]. Recently, we reported that the recombinant Hsp70 applied in a form of hydrogel to mouse melanoma B16 tumour penetrated cancerous tissue, reduced the rate of tumour growth and expanded the survival period of animals [19]. The fact that pure Hsp70 delivered inside a tumour is clinically relevant in anti-cancer therapy prompted us to explore the reaction of the protein with tumour cells in more detail. It was found that the labelled recombinant Hsp70 enters a cell and pulls out its intracellular analogue to a plasma membrane; simultaneously with this exchange the cells become sensitized to the cytotoxic effector cells, as shown with the aid of cytotoxic cell assay. The data of cell transport marker and inhibitor analysis show that the interdependent transport of exo- and endogenous chaperones is performed by several transport pathways, both “classical” and non-classical ones.

## RESULTS

**Fig 1:**
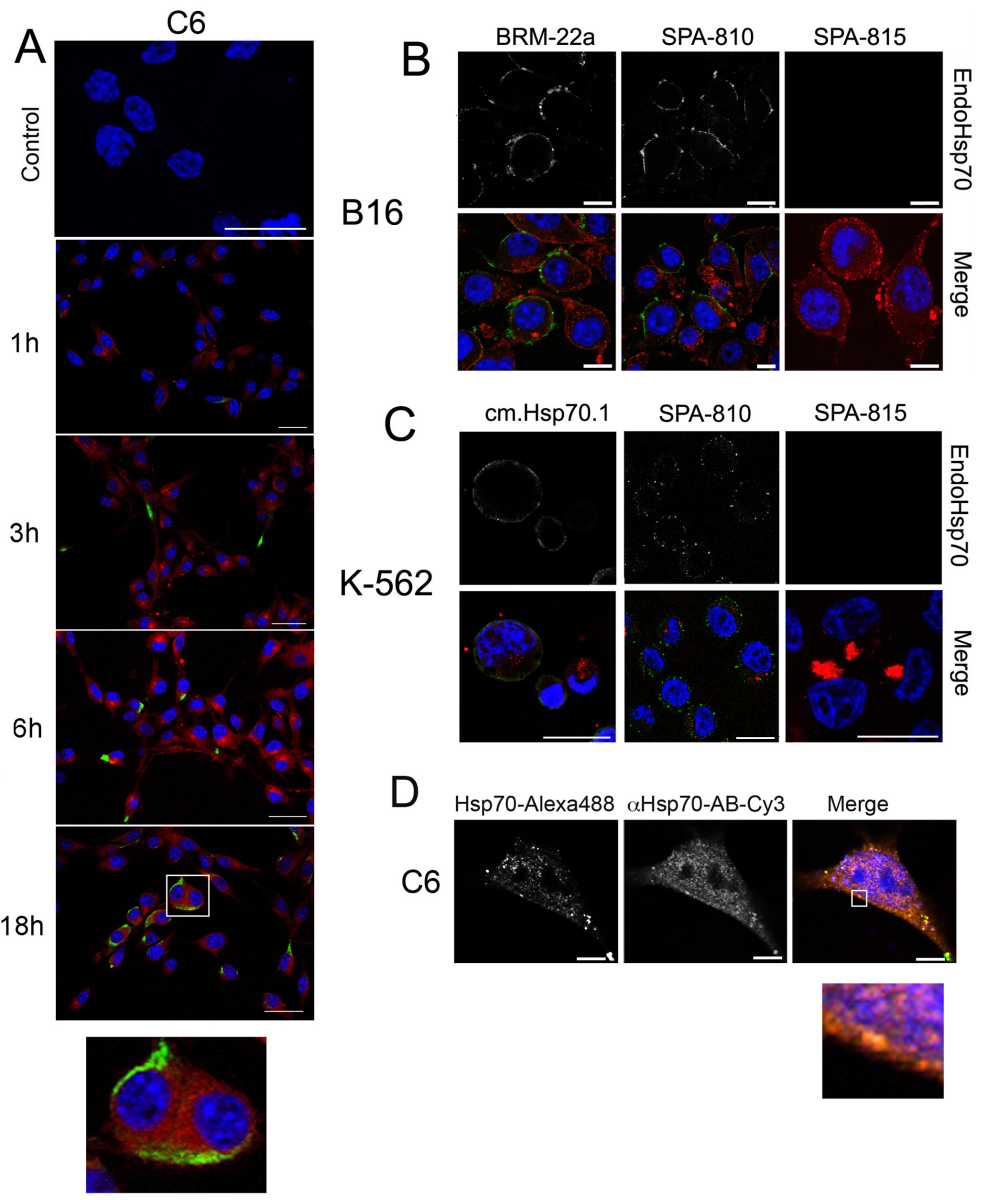
Exogenous Hsp70 introduced in cell culture penetrates inside cells, pushes out own self cellular chaperone A. Confocal microscopy: Hsp70 labelled with Alexa 555 (red) was introduced to C6 glioblastoma cells for the time indicated. Cells were washed, incubated with SPA810 antibody to Hsp70 and, after fixation, stained with Cy2-conjugated anti-mouse antibody (green). Nuclei were stained with DAPI. Scale bar, 10 µm. B,C. Mouse melanoma B16 and K-562 human erythroblastoid leukaemia cells were incubated with Hsp70 labelled with Alexa 555 (red) for 6 hours (K-562) or 18 hours (B16) and further treated with SPA810 antibody to Hsp70 or SPA815 to Hsc70. Merged images presented. Scale bar, 5 µm. Images for DAPI, Cy2 and Alexa555 were obtained using sequential image recording. D. C6 glioblastoma cells were incubated with exo-Hsp70 labeled with Alexa 488 (green) for 18 hours. Following incubation cells were washed, fixed, permeabilized with 0.1% TritonX-100 and stained with SPA 810 antibody (red). Nuclei were stained with DAPI (blue). Scale bar, 1 µm.

The aim of the present study was to explore the reaction of exo-Hsp70 with cancer cells, and we chose several cell lines distinct in their physiology and potential response to effector cells; the lines were rat glioblastoma C6, mouse melanoma B16, erythroleukaemia K-562, U-937 and HL-60 myeloid leukaemia cells. Recombinant Hsp70 conjugated with Alexa Fluor 555 (red) was added to the cell cultures, and its localization was studied using confocal microscopy. The analysis of images showed that the chaperone penetrated inside living C6 cells and this transport took less than 1 h reaching its maximum at 18 h (Fig. 1A). During 3 h of incubation with labelled exo-Hsp70, endogenous chaperone stained with SPA-810 antibody appeared at the cell surface as bright green spots, and the number of such cells increased with time (Fig. 1A). Notably, the green spots did not co-localize with the red staining of exo-Hsp70. Eighteen hours after the beginning of incubation with the exo-Hsp70, cells exhibited powerful and separate staining of green and red colours, indicating that the chaperone pumping machinery functioned in the majority of the C6 cell population (Fig. 1A).

A similar mechanism of Hsp70 exchange functions in B16 mouse melanoma and K-562 human erythroleukaemia cells. As in the case of C6 glioblastoma cells, the exogenous chaperone efficiently penetrates cancer cells within the first 1–6 hours of incubation and this process is associated with the accumulation of endo-Hsp70 close to the cell membrane during the next 12 h (Fig. 1B and C). To prove the different localization of exo-Hsp70 (red) and of its endogenous counterpart in K-562 cells, we stained the latter with two different monoclonal antibodies, SPA-810 and cmHsp70.1 – the latter generated against TKD peptide of Hsp70 expressed on the surface of tumour cells [20]. In cells treated with the exo-Hsp70 cmHsp70.1 antibody stained the whole cell surface while bright red spots of the exogenous chaperone formed large diffuse particles inside the cell (Fig.1B).

Similar data demonstrating exo-Hsp70 penetration and expulsion of the endogenous chaperone were obtained for human myeloid leukaemia HL-60 cells. In these cells part of exo-Hsp70 labelled with Alexa-555 was co-localized with green spots of its endogenous analogue that resulted in yellow staining (supplemental Fig. S1A).

Since Hsc70 cognate protein can also be involved in the chaperone exchange, we treated K-562 and B16 cells with Hsp70-Alexa555 and stained them with two antibodies, SPA-810 and SPA-815, recognizing Hsp70 inducible and Hsc70 constitutive members of Hsp70 family, respectively. Surprisingly, exo-Hsp70 had no effect on Hsc70 distribution within the cell (Fig.1 B,C).

To study mutual spatial arrangement of Hsp70 penetrated C6 cells and its endogenous analogue, we fixed, permeabilized the cells and stained Hsp70 species with SPA-810 antibody. Since secondary antibody was conjugated with Cy-3 dye (red) and exo-Hsp70 was fused with Alexa488 (green) we observed in Merge image partial yellow staining reflecting the presence of exo-Hsp70 in the cytoplasm and beneath the plasma membrane (Fig. 1D). Importantly, cortical layer of membrane was stained in red suggesting the concentration of endo-Hsp70 on the cell surface (Fig. 1D, see insert).

To demonstrate that only Hsp70 can expulse its intracellular analogue, we incubated K-562 cells with 50 g/ml bovine serum albumin conjugated with Alexa555; although the protein also showed a limited penetrative ability, we failed to find any transport of endogenous Hsp70 irrespective of whether the latter was stained by SPA-810 or cmHsp70.1 antibody (Suppl. Fig. S2).

Taken together, these data show that exogenously delivered pure Hsp70 penetrates living cells and induces the migration of the endo-Hsp70 towards cell surface; this process is active and lasts not less than 18 h.

To check whether Hsp70 intra/extracellular transport is able to increase the sensitivity of cancer cells to lymphoid cells, we performed a cytotoxic assay using splenocytes of C3H mice (for C6 and B16 cells) and human peripheral blood mononuclear cells (PBMC) (for K-562, U-937 and HL-60 cells) as effector cells. The level of cell death was estimated as the activity of lactate dehydrogenase occurring in culture medium.

**Fig 2:**
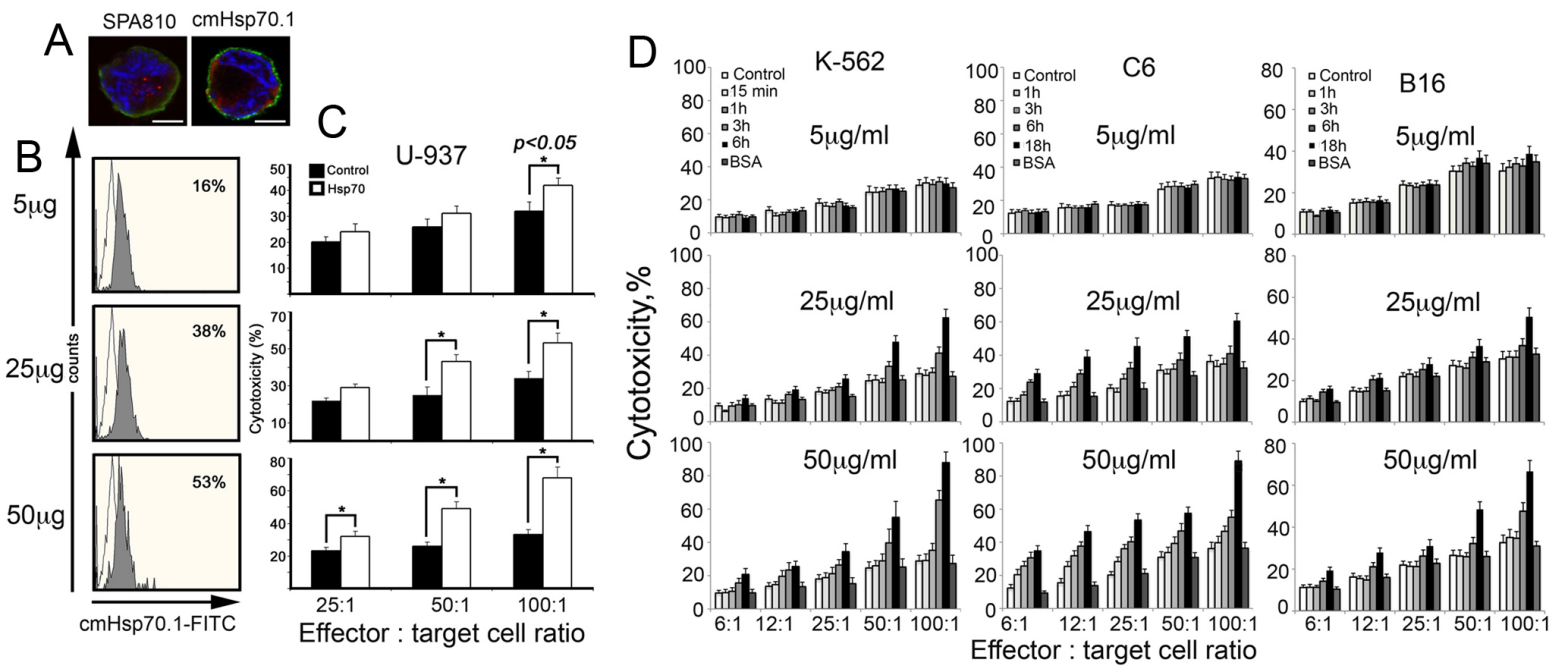
Exogenous Hsp70 introduced in cell culture increases the activity of cytotoxic lymphocytes towards cancer cells of various lines in dose-dependent manner A-C. Human lymphoid leukaemia U-937 cells treated with exogenous Hsp70 expose their own cellular chaperone on cell surface which leads to sensitization to cytotoxic lymphocytes. A. Confocal microscopy: Hsp70 labelled with Alexa 555 (red) was introduced to U-937 for 6 hours. Cells were washed, incubated with antibody to Hsp70 (SPA810 or cmHsp70.1) and, after fixation, stained with Cy2-conjugated anti-mouse antibody (green). Nuclei were stained with DAPI. Images for DAPI, Cy2 and Alexa555 were obtained using sequential image recording. Scale bar, 2,5 μm. B. U937 cells were treated with 5, 20 and 50 μg/ml Hsp70 for 6 hours and stained with cmHsp70.1-FITC mAbs recognizing membrane-bound form of Hsp70. C. Cytotoxicity of PBMCs (effector cells) towards U937 cells (target cells) treated with 5, 20 and 50 μg/ml Hsp70 for 6 hours was estimated as LDH activity in culture medium using Promega CytoTox 96 assay. Untreated U-937 were used as control. The cytolytic activity of PBMCs was assessed at 25:1, 50:1 and 100:1 effector: target cell ratio. D. K-562 human erythroblastoma, C6 rat glioblastoma and B16 mouse melanoma cells were incubated with 5, 25 and 50 mg/ml of recombinant Hsp70 for time ranges indicated and co-cultured with mouse splenocytes isolated from C3H mice and taken in the ratios to target cells as indicated. Cell toxicity was measured as above.

In U-937 human leukaemia cells, the principle of Hsp70 chaperone exchange functions in full accordance with other cell types tested earlier: the pulling out of the endo-Hsp70 was confirmed by its staining with both SPA-810 and cmHsp70.1 antibodies (Fig.2A). Flow cytometry data demonstrated that the elevation of the exo-Hsp70 loading from 5 to 50 µg/ml of medium increased the amount of cells with the endogenous chaperone accumulating at the cell surface from 16% to 53%, suggesting that only a high level of exo-Hsp70 is effective in the extrusion of the its endogenous analogue (Fig.2B). Next we incubated U-937 cells with non-labelled Hsp70 in different concentrations, after which the cells were subjected to PBMC taken in three ratios to target cells. It was found that incubation with the chaperone elevated cytotoxicity two-fold in a step-wise manner (Fig. 2C). Quite similar profiles of the cytotoxicity mediated by exo-Hsp70 were found for the other cell lines, K-562, C6 and B16 cells (Fig.2D). In experiments with C6, K-562 and B16 cells, the degree of cytotoxicity enhancement grew with time; it was low during the first 3–6 h and elevated 2.5-fold up to 18 h of cell reaction with the chaperone. For instance, effector cells started to respond to C6 cells when the latter experienced maximal chaperone cycling, as established by the morphological data, see Fig.1A. Interestingly, the cytotoxicity-enhancing activity of exo-Hsp70 in K-562 erhythroblasts was much greater than that in two other cell lines all treated with the chaperone in the same concentration. The development of cytotoxic activity also proceeded faster towards K-562 cells than towards B16 and C6 cells. In all cases the increase of exo-Hsp70 concentration corresponded to a dose-dependent elevated cytolysis, with the maximal response achieved with 50 µg/ml. In this study bovine albumin employed as a protein control showed no cytotoxicity-enhancing effect.

**Fig 3:**
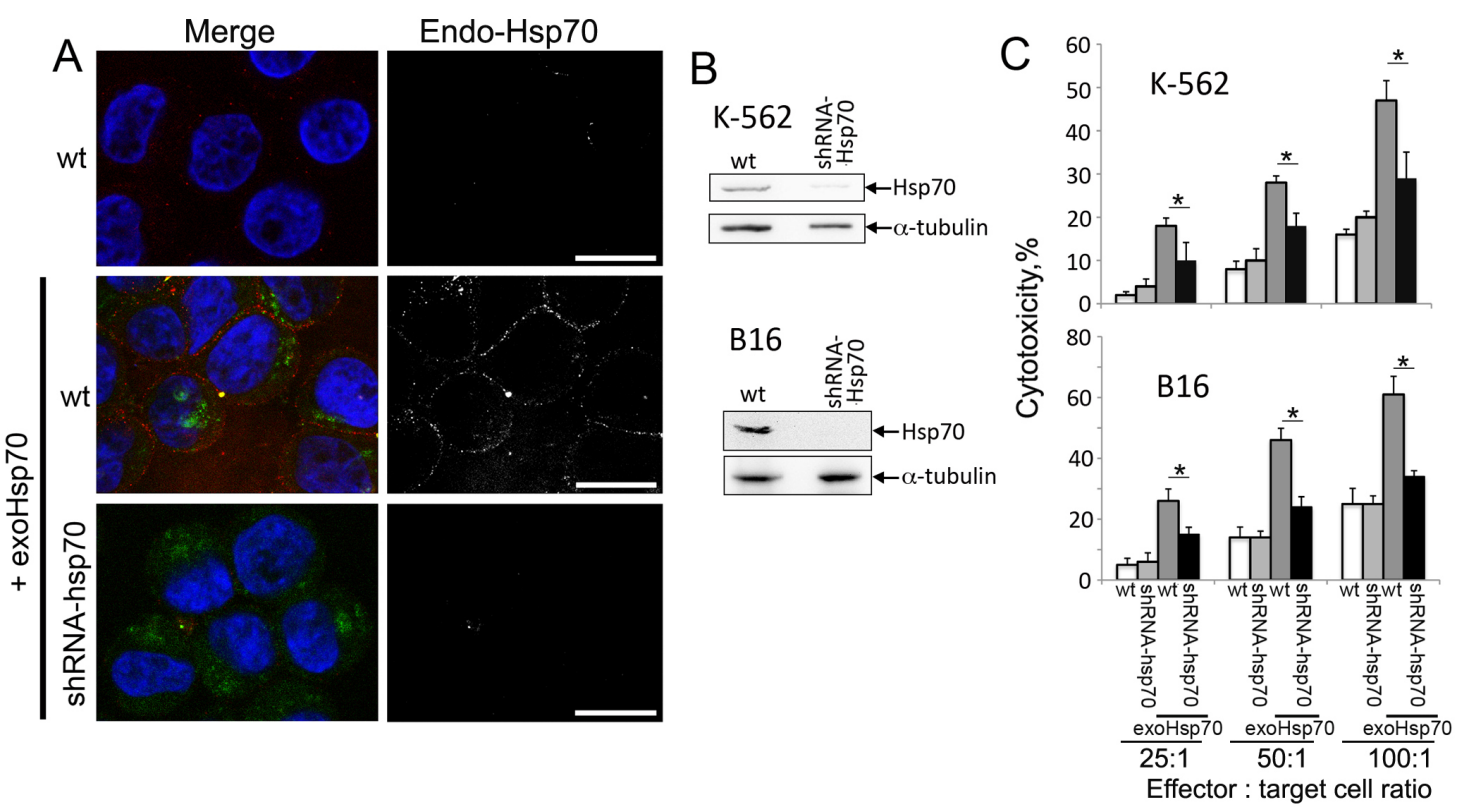
Abrogation of Hsp70 expression reduces the sensitivity of cancer cells towards cytolytic activity of lymphocytes A. Confocal microscopy of K-562 cells incubated with Hsp70-Alexa Fluor®488 (50 µg/ml, *green*) for 6 hours. Membrane-bound Hsp70 was detected with RSIII polyclonal antibodies (*red*). Additionally K-562 were infected with lentivirus construct bearing anti-Hsp70 shRNA cells prior to incubation with exogenous Hsp70 (*lower row*). Nuclei were stained with DAPI (*blue*). Scale bar – 7 µm. B. Western Blotting of K-562 and B16 cells infected with anti-Hsp70 shRNA-encoding lentivirus. Antibody against α-tubulin was employed for loading control. C. Cytotoxic activity of lymphocytes towards B16 or K-562 cells at various effector : target cell ratio. B16wt, K-562wt cells and those infected with lentivirus construct bearing anti-Hsp70 shRNA were incubated with Hsp70 (50 µg/ml) for 18 h.

To check whether the migration of endo-Hsp70 to the cell surface can be a sole factor of cytolysis initiation, we reduced the protein level in K-562 and B16 cells with the aid of lentivirus construct bearing anti-Hsp70 shRNA. The data of immunoblotting proved that the infection was able to efficiently lower the Hsp70 content in the cells of both lines (Fig. 3B). As expected, depletion of endo-Hsp70 caused loss of its expression on K-562 cell surface irrespective of whether they were affected by the exogenous chaperone or not. Meaningfully, the decrease of Hsp70 content in infected cells resulted in 50% reduction of cytotoxic activity towards K-562 and B16 cells treated with 50 g exo-Hsp70 (Fig.3C). When exo-Hsp70 was not applied depletion of endo-Hsp70 in cells of both lines did not lead to increase of the sensitivity of tumour cells to cytolysis (Fig.3C).

**Fig 4:**
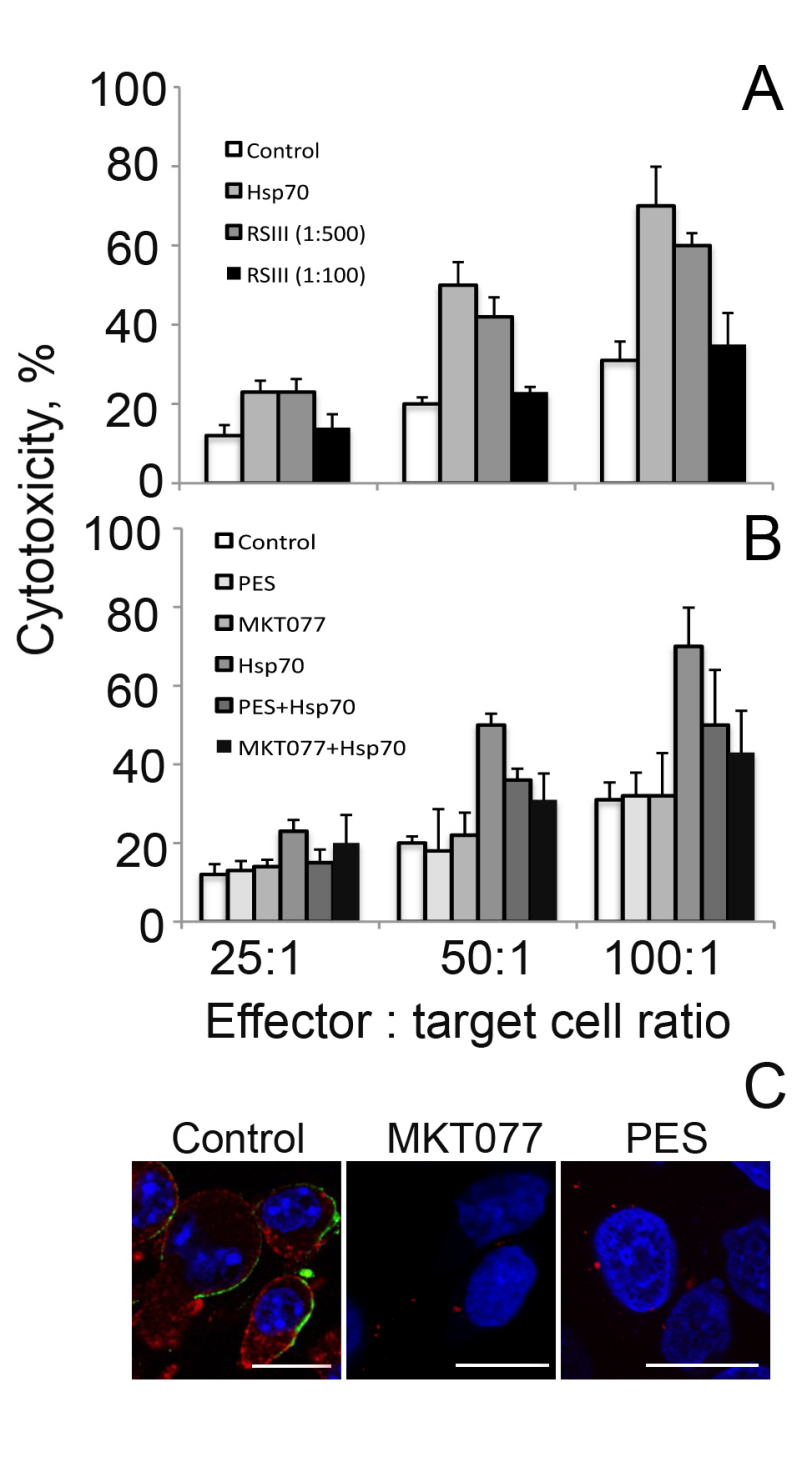
Whole Hsp70 molecule is needed for acquisition of cytotoxic activity A. Cytotoxic activity of lymphocytes towards C6 cells incubated with exogenous Hsp70 (50 µg/ml) for 18 h. During incubation of C6 cells with Hsp70 polyclonal RSIII antibodies were added to the cell medium (dilution 1:100 or 1:500). B. Cytotoxic activity of lymphocytes towards C6 cells incubated with exogenous Hsp70 (50 µg/ml) for 18 h in presence of two factors, PES (2-phenylethynesulfonamide) binding to C-terminal domain and MKT-077 known to interact with ATP-ase N-terminal part of the chaperone molecule. In these experiments C6 cells were incubated with Hsp70 and one of the above factors followed by reaction with lymphocytes in cytotoxicity assay as described above. C. C6 glioblastoma cells incubated with exo-Hsp70-Alexa-555 alone (red) in presence of MKT077 or PES were stained with anti-Hsp70 antibody (green) and fixed. Nuclei were stained with DAPI (*blue*). Scale bar – 5 µm.

Next we decided to inhibit the cytotoxicity-stimulating effect of exo-Hsp70 specifically with the use of a neutralizing antibody. We employed a RSIII polyclonal antibody earlier shown to recognize human recombinant Hsp70 in immunoprecipitation assay. C6 glioblastoma cells were incubated with Hsp70 and the RSIII IgG in two dilutions, 1:100 and 1:500 18 h, washed and incubated for the next 4 h with splenocytes isolated from C3H mice. The incubation of tumour cells with Hsp70 and the specific antibody reduced the level of cytolysis slightly when the antibody was diluted 500 times, whereas cytotoxicity dropped to a control level when the antibody was diluted 100-fold (Fig.4A).

Hsp70 consists of two functionally distinct domains and in order to check whether the whole molecule of the chaperone or its part is necessary for the lymphocyte-stimulating activity we employed two factors: PES (2-phenylethynesulfonamide) binding to C-terminal domain [21] and MKT-077, known to interact with ATP-ase N-terminal part of the chaperone molecule [22]. In these experiments C6 cells were incubated with Hsp70 and one of the above factors, followed by a reaction with mouse splenocytes in various ratios to tumour cells. The results of these experiments show that PES and MKT-077 reduced cytotoxicity-activating power of Hsp70 by 20 and 27% respectively at the effector cell: target ratio of 100:1 (Fig.4B). The factors added in the culture singly did not affect cytotoxicity of lymphocytes suggesting that their effect is related to the inhibition of exo-Hsp70 only (Fig.4B). Furthermore, PES and MKT-077 strongly reduced the ability of exo-Hsp70 to penetrate C6 cells and of endo-Hsp70 to migrate to the cell surface (Fig.4C). We conclude that the whole molecule of the exogenously administered Hsp70 is necessary for eliciting anti-tumour lymphocyte-mediated response.

**Fig 5:**
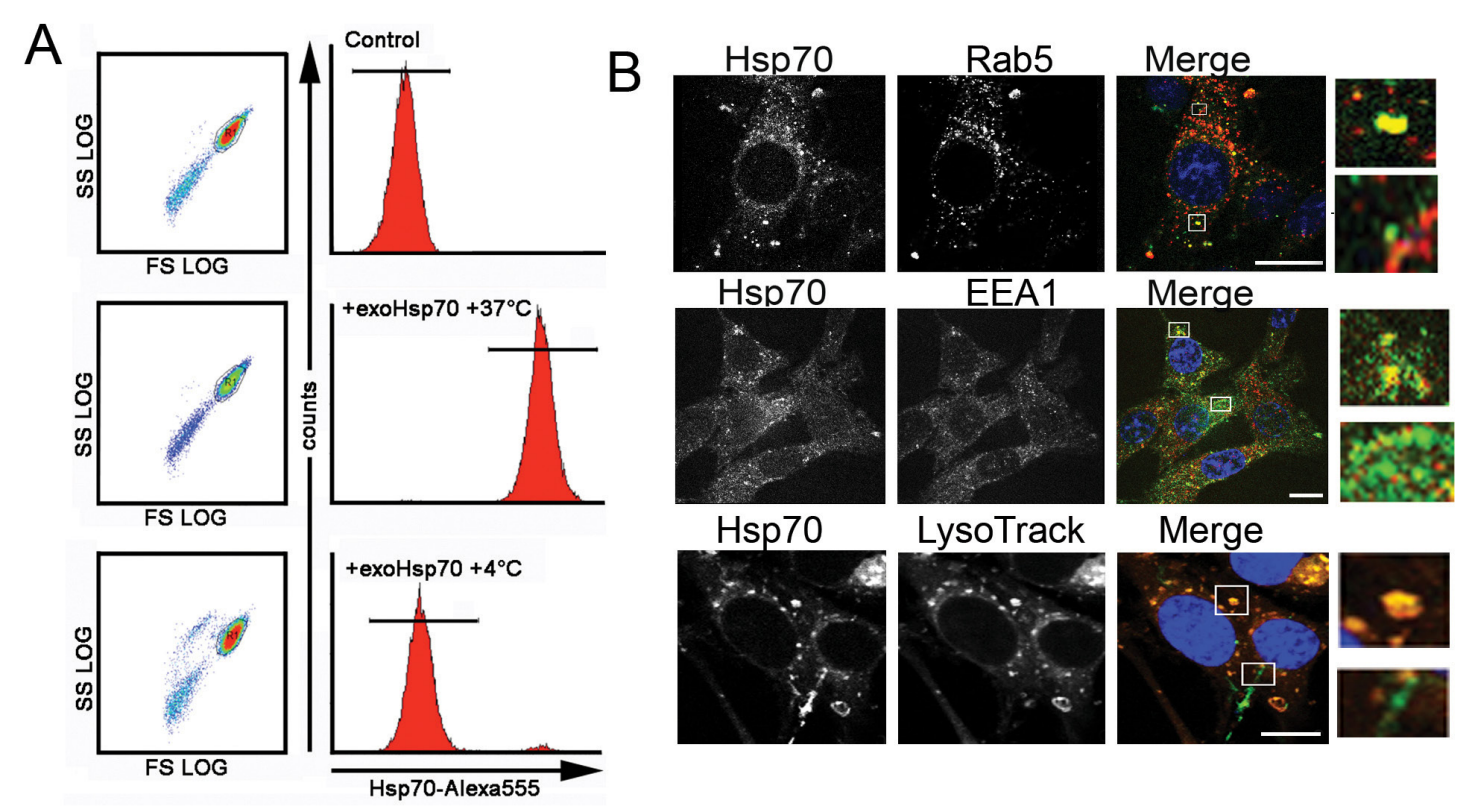
Exo-Hsp70 is internalized by cancer cells employing the mechanisms of active transport A. C6 cells incubated with Hsp70-Alexa Fluor®555 (50 µg/ml) for 18 h at various temperature regimens – 4°C or 37°C – were subjected to flow cytometry analysis. B. Confocal microscopy of C6 cells expressing marker proteins and incubated with Hsp70-Alexa®Fluor 488 (*green*) for 18 h. Upper panel: C6 cells tranfected with rab-5-RFP plasmid (red) after incubation with exo-Hsp70 (green); second line of images, C6 cells fixed, permeabilyzed and stained with anti-EEA-1 antibodies (*red*). Lower panel: lysomes were detected with the help of LysoTracker® (*red*), nuclei stained with DAPI (*blue*). Scale bar – 5 µm. Inserts show patterns of exo-Hsp70 staining co-localized and without vesicular structures.

Generally the data presented here show that the main reason for sensitization of tumour cells to lymphocyte-mediated cytotoxicity is an interdependent migration of Hsp70, exogenous inside a cell and endogenous to the cell surface. To elucidate the features of this migration we checked whether the intracellular transport of exo-Hsp70 was performed using active mechanisms. C6 cells were incubated with Hsp70 labelled with Alexa488 for 18 h at 4°C or 37°C and, after washing, were subjected to flow cytometry. The data show that at 37°C Hsp70 penetrated within practically all cells (99.5%), while at 4°C only a small part of the cell population (0.05%) was able to uptake the protein, suggesting that Hsp70, when entering C6 cells, employs the mechanism of active transport (Fig.5A).

Endocytosis is thought to be the most probable mechanism for protein penetration inside a mammalian cell. To check whether endocytosis is involved in Hsp70 intracellular transport we performed microscopic studies with the aid of specific markers of vesicular transport. First, C6 cells were transfected with rab5-RFP plasmid and incubated with Hsp70-Alexa488 (green) conjugate for 18 h. Second, we stained C6 cells after their reaction with exo-Hsp70 with the antibody against EEA1, a protein whose expression occurs in the process of early endosome fusion [23]. Finally, lysosomes potentially transporting protein cargo were explored in the same cells using LysoTracker dye. We found two intracellular locations of the chaperone entering a cell; one matched with all three vesicle markers, resulting in yellow color in the merge image (Fig 5B, see inserts). The other part of the Hsp70 molecules was found in separate locales that did not coincide with the above markers, suggesting at least two alternatives of Hsp70 intracellular transport.

**Fig 6:**
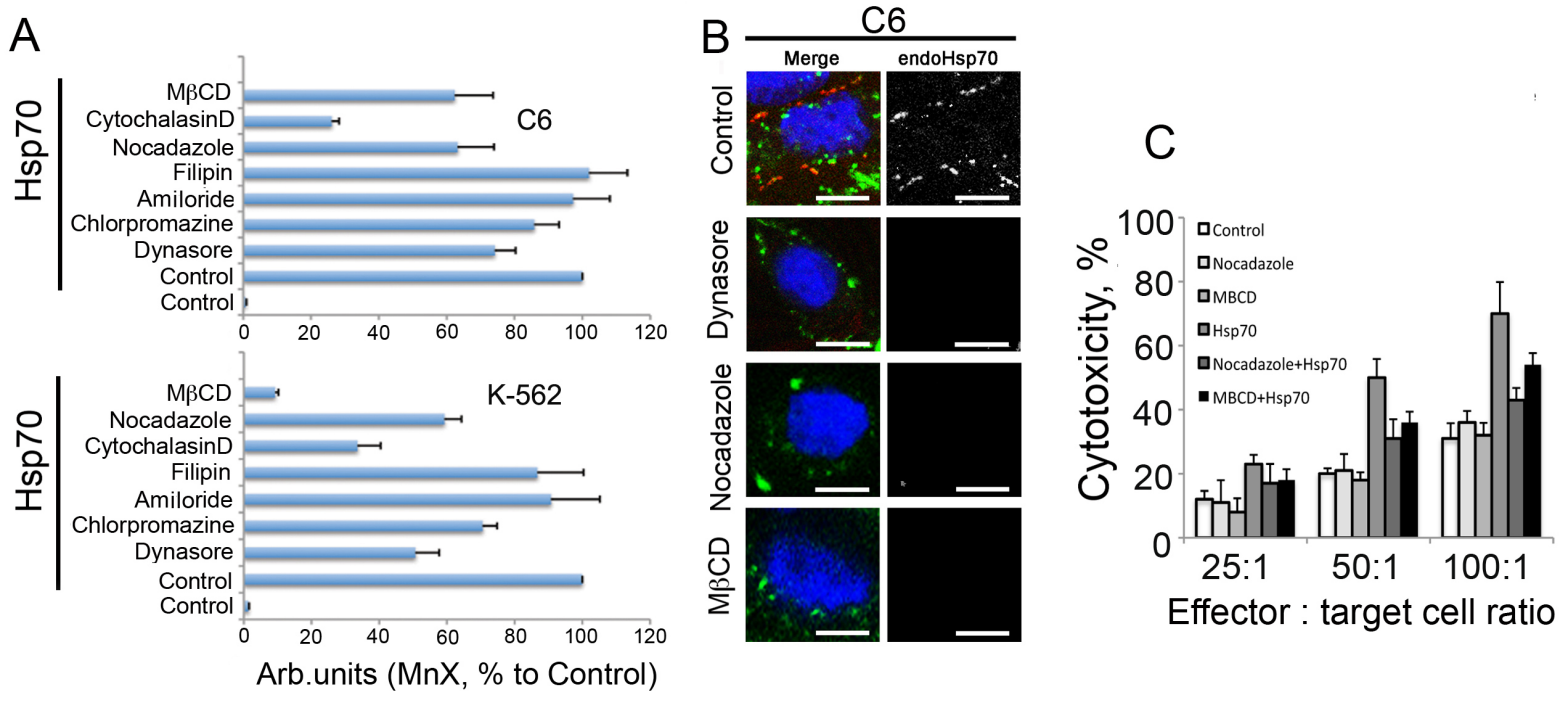
Inhibition of exo-Hsp70 transport reduces self Hsp70 surface exposition and weakens sensitisitation to cytotoxic cells A. Data of flow cytometry. C6 and K-562 cells were incubated with exo-Hsp70 labelled with Alexa488 (50 µg/ml) in the presence of transport inhibitors for 6 h. Data of six independent experiments are summarized. Data presented as mean +SE. B. Confocal microscopy of cells treated with Hsp70 and transport inhibitors. Membrane-bound Hsp70 was detected with RSIII antibodies (*red*) and nuclei were stained with DAPI (*blue*). Scale bar – 2.5 µm. C. Reduction of the exogenous Hsp70 incorporation into cancer cells leads to decreased cytotoxic activity of lymphocytes. Cytotoxicity of lymphocytes towards C6 cells incubated with exo-Hsp70 (50 µg/ml) in presence of nocadazole or methyl-β-cyclodextrin, respectively.

To explore the features of exo-Hsp70 intracellular migration more carefully we performed inhibitor analysis of transport pathways using C6 and K-562 cells as the chaperone targets. For this work we used dynasore, an inhibitor of dynamin-dependent endocytosis; chlorpromazine, known to suppress clathrin-dependent endocytosis; amiloride, a potential inhibitor of macropinocytosis; filipin, which suppresses calveolin-dependent endocytosis; cytohalasine D, which disrupts actin-dependent transport; nocadazole, which inhibits tubulin-dependent endocytosis; and methyl-beta cyclodextrin (MβCD), known to inhibit caveolin-dependent endocytosis and destruct lipid rafts. C6 and K-562 cells were treated with the above factors together with 50 g/ml Hsp70-Alexa488 for 6h and, after washing, were subjected to analysis with the aid of flow cytometer. We found that filipin and amiloride had no effect on the ability of Hsp70 to enter the cells, suggesting that macropinocytosis and caveolar endocytosis are not involved in the transport mechanism in cells of both lines (Fig.6A). Dynasore, the inhibitor of dynamin-dependent endocytosis, suppressed the entry of Hsp70 down to 74.1 +/− 6.2% and 50.7 +/− 7.0% for C6 and К-562 cells, respectively (Fig6A, B). Interestingly, exo-Hsp70, after entering cells, was arrested in the proximity of the plasma membrane, suggesting that dynasore inhibited its cytoplasmic transition. Chlorpromazine had only a modest effect on intracellular transport of Hsp70, reducing the latter to 85.6 +/− 7.4% and 70.6 +/− 4.3% for C6 and К-562 cells respectively (Fig.6A). Inhibitors of cytoskeleton-dependent endocytosis, i.e. nocodazole (tubulin) and cytoholasin D (actin microfilaments), were also shown to reduce the uptake of exogenous chaperone to 63.2 +/− 10.7% (C6 cells) and 59.3 +/− 5.0% (К-562) and to 26.1 +/− 2.1% (C6) and 33.6 +/− 6.9% (К-562), respectively. The employment of MβCD dramatically diminished Hsp70 intracellular transport, especially in K-562 erythroblasts: the residual uptake of Hsp70-Alexa555 constituted only 9.2 +/− 1.0% of the control value for untreated cells. In C6 cells this value comprised 62.3 +/− 7.4% (Fig.6A, B). Since filipin, the specific inhibitor of caveolin-dependent endocytosis did not influence the entry of exo-Hsp70 and MβCD did impede that almost completely we conclude that lipid rafts is an alternative way for the chaperone to cross plasma membrane.

Lastly, to establish whether inhibitors of cellular transport actively suppressing the entry of exo-Hsp70 could affect the expression of intracellular protein on the cell surface, we stained C6 cells incubated with the labelled Hsp70 and three inhibitors including dynasore, nocadazole and MβCD, with antibody to Hsp70. It was shown that all three inhibitors suppressed the transport of the endogenous Hsp70 to a cell surface (Fig.6B). Most important was that concomitantly with the suppressing Hsp70 transport to the cell surface, all three substances reduced the sensitivity of cancer cells to effector splenocytes, as was demonstrated in cytotoxic assay; this inhibition was particularly essential (p<0.01) in C6 cells treated with nocodazole and MβCD (Fig.6D). The inhibitors alone had no effect on cytotoxic activity of splenocytes.

**Fig 7:**
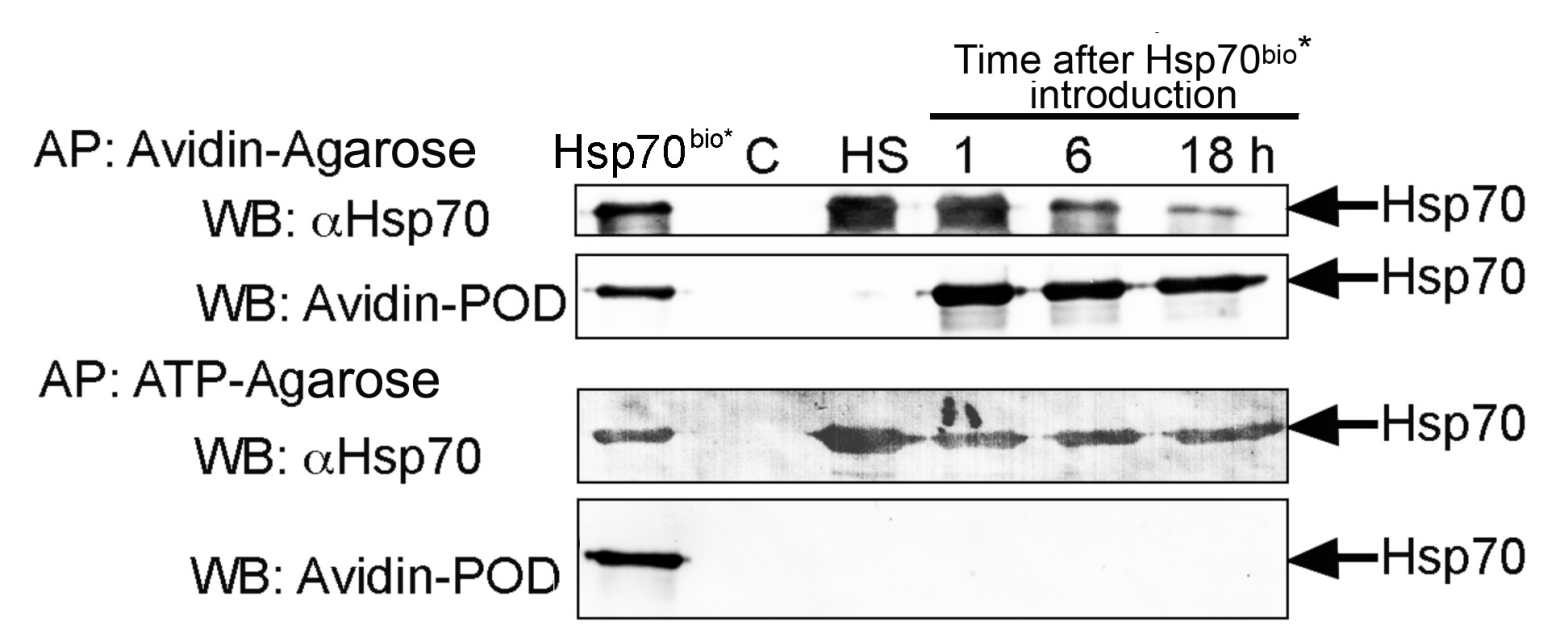
Exogenous Hsp70 provokes its own cycling and release of endogenous chaperone from C6 tumour cells C6 cells (15×10^6^ per probe) were incubated with 50 µg/ml of biotinylated Hsp70 and, after 1, 6 and 18 h, were washed and allowed to export the chaperone into serum-free culture medium for a 90 min period. Culture media were subject to precipitation with NeutrAvidin-agarose (Avidin-Agarose) first, then unbound fractions were precipitated with ATP-agarose (ATP-Agarose). Both sets of eluates from Avidin-Agarose and ATP-Agarose were divided into two parts and subjected to polyacrylamide gel electrophoresis and to immunoblotting; one part of the blots was probed with antibody to Hsp70 and another with Avidin-Peroxidase. C and HS: Hsp70 from the conditioned medium of control and heat-stressed cells (43°C, 30 min) respectively. Hsp70^bio^* – the lane represents the loading of 50 ng of pure biotinylated Hsp70 on the gel.

The intracellular distribution of formerly exo-Hsp70 indicates that at least part of its molecules can be withdrawn by an acceptor cell. According to the data presented in Fig.5B, the exogenous chaperone is not hampered at a cell surface as is the case for the endogenous one. In order to trace the fate of exo-Hsp70 outside a cell we employed the method of trapping the Hsp70 using two affinity media. To discriminate between the exogenously introduced Hsp70 and the self-chaperone, C6 cells were incubated with biotinylated Hsp70 for the time intervals indicated (Fig. 7), washed and were allowed to export the chaperone into serum-free culture media. The conditioned medium was subjected to affinity precipitation with the aid of NeutrAvidin-agarose, and the unbound material was mixed with ATP-agarose gel. Two sets of samples, NeutrAvidin-agarose and ATP-agarose precipitates were analysed with the aid of blotting and staining with Avidin-Peroxidase or anti-Hsp70 polyclonal antibody, recognizing both the rat and human chaperones. The data showed that the export of biotinylated and non-labelled endo-Hsp70 started within 2.5 hours from the beginning of incubation. The release of both proteins lasted for 18 h of observation; however, that of the biotinylated protein became weaker with time (Fig. 7). Leakage of the endo-Hsp70 was comparable to that obtained after heat shock at 43oC (HS). Since no notable leakage of the chaperone was observed from non-treated cells, we concluded that the transport of endo-Hsp70 was induced only by the overloading of cells with the exogenous chaperone or by the cellular chaperone synthesized due to heat stress (Fig. 7). Furthermore, the mortality of C6 cells affected by exo-Hsp70 or heat stress did not exceed 5–7%, thus indicating that mostly living cells had exported the chaperone. The biotinylated BSA control protein did not induce any extracellular transport of endo-Hsp70 (Suppl. Fig. S2). Thus, introduction of Hsp70 into a cancer cell culture leads to its own cycling inside a cell together with the endo-Hsp70. Another notable detail of endo-Hsp70 transport was that, once initiated, it continued at a similar rate during the whole period of observation. We conclude that the exchange between the extracellular and intracellular chaperones occurs actively during at least an 18-hour incubation period of C6 cells with the pure recombinant chaperone.

## DISCUSSION

During last few years a few of newer anti-cancer techniques were reported targeting Hsp70 protective power in tumour cells. One of the latter is based on Hsp70 induction by heat stress and administration of bortezomib, inhibitor of proteasomes; their combined action was shown to provide the exclusively high apoptogenic effect thus suggesting that the protective role of the intracellular Hsp70 be surmounted [24]. In another study Demidenko and coauthors showed that Hsp70 induced by pharmacological blokader of Hsp90 (17-AAG) protected cells from pro-apoptotic factors, but only the cells that are already apoptosis-prone. It make possible to evade Hsp70-mediated protective barrier in anti-cancer therapy [25]. The other approach to exploit the fact of the enhanced amount of the intracellular Hsp70 is to force the latter out of a tumour cell; as we show here this can be performed with the aid of the recombinant Hsp70 delivered into culture of cancer cells. The delivery of the pure chaperone in a tumour was found to be therapeutically relevant in suppressing the growth of B16 mouse melanoma [19], T-cell mouse lymphoma [26]. Since in all these studies Hsp70 alone or in complex with particular tumour antigens was injected systemically or intratumourally, the question is: how does the chaperone induce the loss of tolerance of a cancer cell. This study was carried out to elucidate the basics of recombinant Hsp70's reaction with tumour cells of different origin. The principal finding of these studies was that labelled exo-Hsp70, by a yet unknown mechanism, could force its inner counterpart to leave the cytosol (Fig. 1). Importantly, we observed the phenomenon of Hsp70 cycling in cells of five different lineages: C6 glioblastoma, K-562 human erythroblasts, B16 mouse melanoma (Fig. 1) as well as HL-60 and U-937 human promonocytes (Fig.2A and Suppl. Fig. S1). The capability of endo-Hsp70 to migrate to a cell surface varies depending on the concentration and time of incubation with the exogenously delivered chaperone (Fig. 2). Interestingly, Hsc70 that is the closest analogue of Hsp70 and that is constantly expressed at a high level in cells, was not able to migrate to a cell surface (Fig.1B, C). To our knowledge, this study is the first demonstration of interdependent cycling of Hsp70 in a tumour cell.

The appearance of Hsp70 on a cancer cell surface can become a signal for the recruiting of lymphoid cells able to recognize the specific peptide of the chaperone exposed on the plasma membrane of the tumour cell [27]. Activation of effector cells leads to cytolysis of tumour cells, as shown in numerous in vitro and in vivo simulations [28,29]. Our results also show that exo-Hsp70 added to the cultures elevated cell sensitivity to lymphocytes in a time- and dose-dependent manner (Fig. 2B). In other words, the level of cell lysis was proportional to the concentration of the exogenous chaperone or to the amount of the latter analogue appearing on a cell surface. Application of Hsp70 in a concentration of 5 µg/ml did not result in significant increase in sensitivity of cancer cells to cytotoxic lymphocytes, while when using 50 µg/ml of chaperone we observed a two-fold increase over the control level; the death of the tumour cells reached 90% for C6 and K-562 cells incubated with the chaperone 18 h and 6 h, respectively (Fig 2B).

Our data favor the model in which exo-Hsp70 enters a cancer cell and pushes its intracellular analogue onto a cell surface; finally both proteins leave a cell using unknown mechanisms. To emphasize the role of each Hsp70 species we performed experiments in which their content was affected by different factors. Firstly, the content of endo-Hsp70 was lowered in the K-562 and B16 cells with the aid of lentiviral constructs carrying specific anti-Hsp70 shRNA. The infection reduced the level of endo-Hsp70 and its amount on a cell surface, whereas the efficacy of intracellular transport of exo-Hsp70 was not affected (Fig.3A). This confirms our suggestion that endo-Hsp70 rather than exo-Hsp70 is delayed at the cell surface. Most important was that a lack of endo-Hsp70 expression at the cell surface caused approximately a two-fold decrease in sensitivity to cytotoxic lymphocytes in K-562 and B16 cells (Fig. 3C). Interestingly, in B16 cells, in which Hsp70 level was reduced to undetectable levels by Western blotting (Fig.3B), inhibition of cell sensitivity to cytotoxic lymphocytes was lowered almost to the control level (Fig.3C).

Secondly, a full-size Hsp70 molecule is needed to cause the powerful response of cytotoxic lymphocytes to tumour cells. Incubation of C6 cells with Hsp70 in the presence of specific polyclonal antibodies led to an almost complete abolishment of cell tolerance to cytotoxic lymphocytes (Fig. 4A). In order to reveal the part of the chaperone molecule responsible for anti-tumour activity we used specific binders of Hsp70 – PES binding C-terminal domain [21] and MKT-077 known to interact with ATP-ase N-terminal part of the chaperone molecule [22]. Incubation of C6 cells with Hsp70 in the presence of these binders has reduced the cytotoxicity of lymphocytes, suggesting that the full molecule of exo-Hsp70 is obligatory for the anti-tumour effect (Fig. 4B). Surprisingly we have not observed any influence of both compounds on the response of target C6 cells to cytotoxic lymphocytes without exo-Hsp70 treatment. One can hypothesize that in non-treated cells the amount of intracellular Hsp70 in a form necessary for its surface presentation is very low to induce the NK cell activity. The constraint of a full molecule for the proper function of Hsp70 has been established in many studies, though even partial fastening of the protein molecule is effective; this gives rise to applications of low molecule weight binders of Hsp70 in models of neurodegenerative pathologies, as well as in tumour models both in vivo and in vitro (see for a review [30]).

The events occurring as a result of incubation of tumour cells with the recombinant Hsp70 include (i) the entry of the latter and its distribution within the cytoplasm and other locales, (ii) slow migration of endo-Hsp70 to a cell surface and (iii) the release of molecules of both types to an extracellular matrix. Since these events are tightly linked to the elevation of cell sensitivity to cytotoxic lymphocytes, we explored the transport of both protein species in more detail.

It is thought that the most probable way for a protein to penetrate within the living cell is endocytotic transport, which can occur by clathrin-dependent internalization, clathrin-independent internalization through caveolae and clathrin- and caveolin-independent internalization [31,32]. Studying the route of exo-Hsp70 inside a cell, we found that (i) this process was active and fully suppressed when the cells were incubated at +4oC (Fig. 5A), (ii) macropinocytosis and caveolar endocytosis do not contribute to chaperone uptake, and (iii) a large part of exogenously administered Hsp70 employs endocytotic pathway for transport (Fig. 5B). We found a part of exo-Hsp70 co-localized with the markers of early endosomes, Rab5 and EEA1. Exo-Hsp70 was found also in association with lysosomal marker (Fig. 5B). The results of inhibitory analysis (Fig.6A) confirmed that the import of exo-Hsp70 may be performed with the contribution of clathrin-dependent endocytosis mediated by receptors with weak affinity. Among the latter are SREC1, Lox1 and CD40 receptors known to be present on a surface of a variety of cells [33,34]. Recently we have shown that CD40 on the surface of C6 glioblastoma cells is a target for Hsp70 linked to magnetic nano-particles [35].

The appearance of exo-Hsp70 in late endosomes may signify its involvement as a cargo in the process of early endosome fusion, with subsequent transition of the protein molecule to the intralumenal vesicles [36]. The major part of late endosomes fuse with lysosomes and their content undergoes proteolytic degradation. Another part of late endosomes with multiple intralumenal vesicles inside can be secreted as exosomes to the extracellular environment upon fusion of late endosome with plasma membrane. The alternative way for an endosome's protein cargo is its release together with the content of intralumenal vesicules to the cytosol [37]. Our data show that the two latter events can occur during the uptake of exo-Hsp70 by C6 cells (Fig. 5B). First, the part of exo-Hsp70 molecules was not found associated with vesicular structures and second, high quantities of biotinylated exo-Hsp70 were actively releasing from C6 cells. Thus we suggest that the pool of the extracellular Hsp70 includes molecules exported by a classical, endocytotic pathway as well as by secretory lysosomes or via lipid rafts [38,29].

There are two observations that may be of interest in view of the possible clinical application of the data. Firstly, biotinylated exo-Hsp70, soon after its admission to a cell, starts leaking from the latter; on its way to and back, the protein does not remain on a cell surface, suggesting that it plays the role of a pusher rather than a regulator of effector cell activity. Moreover, the release of exo-Hsp70 has a tendency to reduce with time and decreases to a very low level at the point of 18 h when the maximal cytotoxic effect of effector cells is observed (Fig.7). Secondly, endo-Hsp70 starts leaking simultaneously with its extracellular counterpart and the release is maintained at the constant level within 18 h and even longer (data not shown). However, the maximal cytotoxic effect of cytotoxic lymphocytes towards C6 cells was observed at the period 18 h after the beginning of the incubation of cells with exo-Hsp70, i.e. when the maximal amount of endo-Hsp70 was exposed at the cell surface (Fig.1A). We suggest that endo-Hsp70 accumulated at the plasma membrane plays a major role in attracting cytotoxic effector as the primary anti-tumour response. The leaking part of endo-Hsp70 molecules, probably in complex with the appropriate antigens and packed into exosomes, may play an immunomodulatory role in the generation of the specific anti-cancer response [39].

Earlier heat shock treatment of cancerous cells, accompanied by the powerful efflux of the chaperone, was found to induce anti-tumour activity [28]. The data presented here show that the intra-tumoural administration of Hsp70 could be an alternative to targeted hyperthermia in treating cancer. Similar data were obtained when the patients were vaccinated with autologous Hsp70 which, at the cellular level, led to the initiation of crosstalk between dendritic and NK cells through the activation of their respective molecules, MHC class I chain-related protein MICA and NKG2D ligand [40]. Another important function of both the exo-Hsp70 and of the endo-Hsp70 released from cancer cells may be the stimulation of MyD88/IRAK4-dependent pathway [41]. These recent data show that Hsp70 released into the extracellular milieu under the influence of a pure chaperone, as in our case, possesses all the activities required to stimulate non-specific and probably also specific immunities. Since the parameters of the cycling are similar in various cancer cells, the chaperone administered intratumourally can be a relatively inexpensive substitute for other immunotherapeutic technologies, especially in the case of chronic and hard malignancies.

## MATERIALS AND METHODS

### Cells

The C6 rat glioma cells, U-937 human lymphoid leukaemia and human erythroid leukaemia K-562 were obtained from the Russian Cell Culture Collection at the Institute of Cytology RAS (St. Petersburg, Russia). Mouse melanoma B16 F10 cells were kindly provided by Prof. L. Sistonen (Turku Centre for Biotechnology, Finland) [42]. C6 cells were grown in DMEM/F12, U-937 and K-562 cells in RMPI-1640 and B16 cells in DMEM media; all were supplemented with 10% foetal bovine serum, 2 mM L-glutamine, 100 U/ml penicillin and 0.1 mg/ml streptomycin. All of these media and sera were purchased in PanEco, Russia.

### Proteins

Recombinant human Hsp70 was purified from bacteria transformed with a pMSHsp70 plasmid, as described elsewhere [43]. Hsp70 solution was further cleaned by incubation with Polymyxin B-agarose gel (Sigma-Aldrich, USA) and sterilized by filtration through a 0.2 µm filter (Millipore). According to the E-Toxate assay (Sigma-Aldrich, USA), the level of lipopolysaccharide in the final Hsp70 preparation was lower than 0.1 MU/ml. For microscopy Hsp70 was conjugated to Alexa555 dye (Invitrogen) according to the manufacturer's protocol. For biochemical experiments, Hsp70 was biotinylated using succinimide-NHS-biotin (Sigma-Aldrich, USA). The control protein, bovine serum albumin (BSA), was labelled as above. In some experiments Hsp70 was incubated with a specific RSIII polyclonal antibody generated in our laboratory. To understand which domain of Hsp70 is responsible for its intracellular transport we incubated Hsp70 before introducing with C-terminal binder PES (25mM, Sigma) or N-terminal binder MKT077 (25 mM, Sigma).

### Flow cytometry

U-937 lymphoid leukaemia cells were incubated with 5, 20 and 50 μg/ml of Alexa-555 labelled Hsp70 6 h at 37oC, 6% CO2 and 90% humidity. After incubation the cells were washed twice with ice-cold phosphate buffered saline (PBS) and stained with cmHsp70.1-FITC monoclonal antibodies at 4o C for 30 min to detect the chaperone localized to a cell membrane [20]. After two washes with ice-cold PBS, viable (propidium-iodide negative) cells were gated and analysed with the aid of Cytomics FC500 (Beckman Coulter, USA) flow cytometer.

To carry out inhibitor analysis of Hsp70 penetration, K-562 human erythroblastoma cells and C6 were incubated with 50 µg/ml Alexa-488 labelled Hsp70 in the presence of 100µM dynasore, an inhibitor of dynamin-dependent endocytosis; 10 µM chlorpromazine, known to suppress clathrin-dependent endocytosis; 50µM amiloride, established to inhibit pinocytosis; 10µM filipin, which suppresses calveolin-dependent endocytosis; 5µM cytohalasine D, which disrupts actin-dependent transport; 2µM nocadazole, which inhibits tubulin-dependent endocytosis; and 5µM methyl-beta cyclodextrin, known to destruct lipid rafts. After two washes with PBS, viable cells were gated and analysed with the aid of Coulter Epics XL (Beckman Coulter, USA) flow cytometer using laser 488 nm.

### Confocal Microscopy

C6 and K652 cells were allowed to settle on poly-L-lysine-coated glass slides. Cells were incubated with Alexa555-labelled Hsp70 for 1, 3, 6 and 18 h at 37 °C. K-562 cells were incubated for 6 h, B16 and C6 cells for 18 h. After incubation with the proteins, the cells were washed with ice-cold PBS, incubated with monoclonal antibodies SPA 810 (StressMarq) for 40 min and fixed with 4% paraformaldehyde in PBS followed by incubation with secondary Alexa488-conjugated goat anti-mouse antibody (Invitrogen). To detect surface Hsp70 in K-562 cells, cmHsp70.1-FITC antibody (Stangl et al., 2011) were used as well. Nuclei were stained with 4′,6-diamidino-2-phenylindole (DAPI).

To obtain a morphological view of inhibitor analysis of exo-Hsp70 intracellular transport, C6 cells were incubated with 50 µg/ml Alexa-488 labelled Hsp70 in the presence of 100µM dynasore, 50µM amiloride and 5µM methyl-beta cyclodextrin, as described above, for 6 hours. Cells were washed twice with ice-cold PBS and incubated with polyclonal anti-Hsp70 RIII antibody, then fixed with 4% pharmaldehyde, followed by incubation with secondary Alexa555-conjugated goat anti-mouse antibody (Invitrogen).

The contribution of the endocytosis pathway to Hsp70 entry into the tumour cell was assessed with the use of C6 glioblastoma cells transfected with plasmid containing rab5-RFP gene (gift of Dr. Oleg Demidov, INSERM, France) and then incubated with 50 µg/ml of Hsp70 labelled with Alexa488. In the other experimental setting C6 cells were first incubated with Hsp70 labelled with Alexa488 for 18 h and then, after careful washing, lysosomes were stained with the aid of LysoTracker® Deep Red Fluorescence (Molecular Probes®) according to manufacturer protocol. Also, C6 cells incubated with Alexa488-labelled Hsp70 were fixed with 4% paraformaldehyde in PBS, incubated with 0.1% Triton X-100 for 30 min and then stained with anti-EE1A antibody (Sigma) and secondary Alexa555-labelled antibody.

Fluorescence images were captured with the use of Leica TCS SP2 confocal microscope (Leica, Germany). To avoid possible cross-interference of various fluorochromes, images for DAPI, Cy2 and Alexa555 were acquired using the sequential image recording method.

### Analysis of Hsp70 Release by Living Cells

C6 rat glioblastoma cells (1,5×107 in each probe) were incubated with 50 µg/ml biotinylated Hsp70, and after 1, 6 and 18 h, the cells were washed with PBS and placed into serum-free media for the next 90 min. After precipitating the cells, the medium was supplemented with 0.1% Tween-20 to destroy possible exosomes and with Tris-HCl buffer pH 7.5 and MgCl2 (final concentrations 20 mM and 5 mM, respectively). The first cycle of affinity precipitation was performed by adding NeutrAvidin-agarose gel slurry (Pierce) to each sample of conditioned medium. The affine resin was washed with 20 mM Tris-HCl, pH 7.5, 5 mM MgCl2, 0.1% Tween-20, mixed with an equal volume of 2-fold buffer for electrophoretic samples containing 2% sodium dodecylsulfate. Unbound NeutrAvidin-agarose material was mixed with the ATP-agarose gel (Sigma-Aldrich) and the mixture was rotated overnight at 4°C. The gel probes were subjected to treatment with SDS as described above. After electrophoresis in 11% polyacrylamide gel, the protein bands were transferred to Immobilon membrane (Millipore, USA) that was stained alternatively with Avidin-Peroxidase (Pierce, USA) or RS-III anti-Hsp70 polyclonal antibody generated in our laboratory and known to recognize human, rat and mouse Hsp70.

### Cytotoxicity Assay

C6 rat glioblastoma, B16 mouse melanoma and K-562 human erythroblasts employed as target cells were treated with 50 mg/ml of recombinant Hsp70 or control protein, BSA, for the indicated time periods. The effector cells (splenocytes) were isolated from rat and mice spleens to be further employed with C6 and B16 cells, respectively; the incubation of effector-target cells in different ratios (as indicated in the text) lasted 4 h. For human leukaemia K-562 cells, we employed human peripheral blood mononuclear cells isolated with the aid of gradient centrifugation using Histopaque-1.077 (Sigma-Aldrich, USA). To determine the amount of dying target cells, the CytoTox 96 non-radioactive cytotoxicity assay (Promega, USA) was performed according to the manufacturer's protocol.

### Statistics

Observations are generally reported as mean±SE. One- or two-tailed unpaired Student's t tests were used to evaluate the differences between the control and treatment groups; differences were considered to be statistically significant when p<0.05.

## SUPPLEMENTARY FIGURES



## References

[1] Calderwood SK, Ciocca DR (2008). Heat shock proteins: stress proteins with Janus-like properties in cancer. Int J Hyperthermia.

[2] Gastpar R, Gross C, Rossbacher L, Ellwart J, Riegger J, Multhoff G (2004). The cell surface-localized heat shock protein 70 epitope TKD induces migration and cytolytic activity selectively in human NK cells. J Immunol.

[3] Sapozhnikov AM, Gusarova GA, Ponomarev ED, Telford WG (2002). Translocation of cytoplasmic HSP70 onto the surface of EL-4 cells during apoptosis. Cell Prolif.

[4] Ponomarev E, Tarasenko TN, Sapozhnikov AM (2000). Splenic cytotoxic cells recognize surface HSP70 on culture-adapted EL-4 mouse lymphoma cells. Immunol Lett.

[5] Moroi Y, Mayhew M, Trcka J, Hoe MH, Takechi Y, Hartl FU, Rothman JE, Houghton AN (2000). Induction of cellular immunity by immunization with novel hybrid peptides complexed to heat shock protein 70. Proc Natl Acad Sci USA.

[6] Figueiredo C, Wittmann M, Wang D, Dressel R, Seltsam A, Blasczyk R, Eiz-Vesper B (2009). Heat shock protein 70 (HSP70) induces cytotoxicity of T helper cells. Blood.

[7] Srivastava P (2002). Roles of heat-shock proteins in innate and adaptive immunity. Nat Rev Immunol.

[8] Asea A, Rehli M, Kabingu E, Boch JA, Bare O, Auron PE, Stevenson MA, Calderwood SK (2002). Novel signal transduction pathway utilized by extracellular HSP70: role of toll-like receptor (TLR) 2 and TLR4. J Biol Chem.

[9] Evdonin AL, Guzhova IV, Margulis BA, Medvedeva ND (2006). Extracellular heat shock protein 70 mediates heat stress-induced epidermal growth factor receptor transactivation in A431 carcinoma cells. FEBS Lett.

[10] Sherman M, Multhoff G (2007). Heat shock proteins in cancer. Ann NY Acad Sci.

[11] Guzhova I, Kislyakova K, Moskaliova O, Fridlanskaya I, Tytell M, Cheetham M, Margulis B (2001). In vitro studies show that Hsp70 can be released by glia and that exogenous Hsp70 can enhance neuronal stress tolerance. Brain Res.

[12] Novoselova TV, Margulis BA, Novoselov SS, Sapozhnikov AM, van der Spuy J, Cheetham ME, Guzhova IV (2005). Treatment with extracellular HSP70/HSC70 protein can reduce polyglutamine toxicity and aggregation. J Neurochem.

[13] Arispe N, Doh M, Simakova O, Kurganov B, De Maio A (2004). Hsc70 and Hsp70 interact with phosphatidylserine on the surface of PC12 cells resulting in a decrease of viability. FASEB J.

[14] Murshid A, Theriault J, Gong J, Calderwood SK (2011). Investigating receptors for extracellular heat shock proteins. Methods Mol Biol.

[15] Massa C, Guiducci C, Arioli I, Parenza M, Colombo MP, Melani C (2004). Enhanced efficacy of tumor cell vaccines transfected with secretable hsp70. Cancer Res.

[16] Wang W, Ji W, Hu H, Ma J, Li X, Mei W, Xu Y, Hu H, Yan Y, Song Q, Li Z, Su C (2014). Survivin promoter-regulated oncolytic adenovirus with Hsp70 gene exerts effective antitumor efficacy in gastric cancer immunotherapy. Oncotarget.

[17] Geng H, Zhang GM, Xiao H, Yuan Y, Li D, Zhang H, Qiu H, He YF, Feng ZH (2006). HSP70 vaccine in combination with gene therapy with plasmid DNA encoding sPD-1 overcomes immune resistance and suppresses the progression of pulmonary metastatic melanoma. Int J Cancer.

[18] Ito A, Matsuoka F, Honda H, Kobayashi T (2004). Antitumor effects of combined therapy of recombinant heat shock protein 70 and hyperthermia using magnetic nanoparticles in an experimental subcutaneous murine melanoma. Cancer Immunol Immunother.

[19] Abkin SV, Pankratova KM, Komarova EY, Guzhova IV, Margulis BA (2013). Hsp70 chaperone-based gel composition as a novel immunotherapeutic anti-tumor tool. Cell Stress Chaperones.

[20] Stangl S, Gehrmann M, Riegger J, Kuhs K, Riederer I, Sievert W, Hube K, Mocikat R, Dressel R, Kremmer E, Pockley AG, Friedrich L, Vigh L, Skerra A, Multhoff G (2011). Targeting membrane heat-shock protein 70 (Hsp70) on tumors by cmHsp70. 1 antibody. Proc Natl Acad Sci USA.

[21] Balaburski GM, Leu JI, Beeharry N, Hayik S, Andrake MD, Zhang G, Herlyn M, Villanueva J, Dunbrack RL, Yen T, George DL, Murphy ME (2013). A modified HSP70 inhibitor shows broad activity as an anticancer agent. Mol Cancer Res.

[22] Rousaki A, Miyata Y, Jinwal UK, Dickey CA, Gestwicki JE, Zuiderweg ER (2011). Allosteric drugs: the interaction of antitumor compound MKT-077 with human Hsp70 chaperones. J Mol Biol.

[23] Mills IG, Jones AT, Clague MJ (1999). Regulation of endosome fusion. Mol Membr Biol.

[24] Neznanov N, Komarov AP, Neznanova L, Stanhope-Baker P, Gudkov AV (2011). Proteotoxic stress targeted therapy (PSTT): induction of protein misfolding enhances the antitumor effect of the proteasome inhibitor bortezomib. Oncotarget.

[25] Demidenko ZN, Vivo C, Halicka HD, Li CJ, Bhalla K, Broude EV, Blagosklonny MV (2006). Pharmacological induction of Hsp70 protects apoptosis-prone cells from doxorubicin: comparison with caspase-inhibitor- and cycle-arrest-mediated cytoprotection. Cell Death Differ.

[26] Kumar S, Deepak P, Kumar S, Kishore D, Acharya A (2009). Autologous Hsp70 induces antigen specific Th1 immune responses in a murine T-cell lymphoma. Immunol Invest.

[27] Gastpar R, Gehrmann M, Bausero MA, Asea A, Gross C, Schroeder JA, Multhoff G (2005). Heat shock protein 70 surface-positive tumor exosomes stimulate migratory and cytolytic activity of natural killer cells. Cancer Res.

[28] Multhoff G (2009). Activation of natural killer cells by heat shock protein 70. 2002. Int J Hyperthermia.

[29] Guzhova IV, Shevtsov MA, Abkin SV, Pankratova KM, Margulis BA (2013). Intracellular and extracellular Hsp70 chaperone as a target for cancer therapy. Int J Hyperthermia.

[30] Patury S, Miyata Y, Gestwicki JE (2009). Pharmacological targeting of the Hsp70 chaperone. Curr Top Med Chem.

[31] Gould GW, Lippincott-Schwartz J (2009). New roles for endosomes: from vesicular carriers to multi-purpose platforms. Nat Rev Mol Cell Biol.

[32] Schwartz V, Krüttgen A, Weis J, Weber C, Ostendorf T, Lue H, Bernhagen J (2012). Role for CD74 and CXCR4 in clathrin-dependent endocytosis of the cytokine MIF. Eur J Cell Biol.

[33] Calderwood SK, Mambula SS, Gray PJ (2007). Extracellular heat shock proteins in cell signaling and immunity. Ann NY Acad Sci.

[34] Becker T, Hartl FU, Wieland F (2002). CD40, an extracellular receptor for binding and uptake of Hsp70-peptide complexes. J Cell Biol.

[35] Shevtsov MA, Yakovleva LY, Nikolaev BP, Marchenko YY, Dobrodumov AV, Onokhin KV, Onokhina YS, Selkov SA, Mikhrina AL, Guzhova IV, Martynova MG, Bystrova OA, Ischenko AM, Margulis BA (2014). Tumor targeting using magnetic nanoparticle Hsp70 conjugate in a model of C6 glioma. Neuro-Oncology.

[36] van Meel E, Klumperman J (2008). Imaging and imagination: understanding the endo-lysosomal system. Histochem Cell Biol.

[37] Huotari J, Helenius A (2011). Endosome maturation. EMBO J.

[38] Mambula SS, Calderwood SK (2006). Heat shock protein 70 is secreted from tumor cells by a nonclassical pathway involving lysosomal endosomes. J Immunol.

[39] Lv LH, Wan YL, Lin Y, Zhang W, Yang M, Li GL, Lin HM, Shang CZ, Chen YJ, Min J (2012). Anticancer drugs cause release of exosomes with heat shock proteins from human hepatocellular carcinoma cells that elicit effective natural killer cell antitumor responses in vitro. J Biol Chem.

[40] Qiao Y, Liu B, Li Z (2008). Activation of NK cells by extracellular heat shock protein 70 through induction of NKG2D ligands on dendritic cells. Cancer Immun.

[41] Mizukami S, Kajiwara C, Tanaka M (2012). Kaisho T, Udono H. Differential MyD88/IRAK4 requirements for cross-priming and tumor rejection induced by heat shock protein 70-model antigen fusion protein. Cancer Sci.

[42] Nagy E, Balogi Z, Gombos I, Akerfelt M, Björkbom A, Balogh G, Török Z, Maslyanko A, Fiszer-Kierzkowska A, Lisowska K, Slotte PJ, Sistonen L, Horváth I, Vígh L (2007). Hyperfluidization-coupled membrane microdomain reorganization is linked to activation of the heat shock response in a murine melanoma cell line. Proc Natl Acad Sci USA.

[43] Guzhova IV, Lazarev VF, Kaznacheeva AV, Ippolitova MV, Muronetz VI, Kinev AV, Margulis BA (2011). Novel mechanism of Hsp70 chaperone-mediated prevention of polyglutamine aggregates in a cellular model of Huntington disease. Hum Mol Genet.

