# Supplementary material for: Exogenously delivered heat shock protein 70 displaces its endogenous analogue and sensitizes cancer cells to lymphocytes-mediated cytotoxicity
Source: Oncotarget. 2014 Mar 22;5(10):3101–14. doi: 10.18632/oncotarget.1820 (PMC4102795; doi:10.18632/oncotarget.1820)
Supplement: Supplementary file 1 [file oncotarget-05-3101-s001.pdf]

Supplemental Figure S1

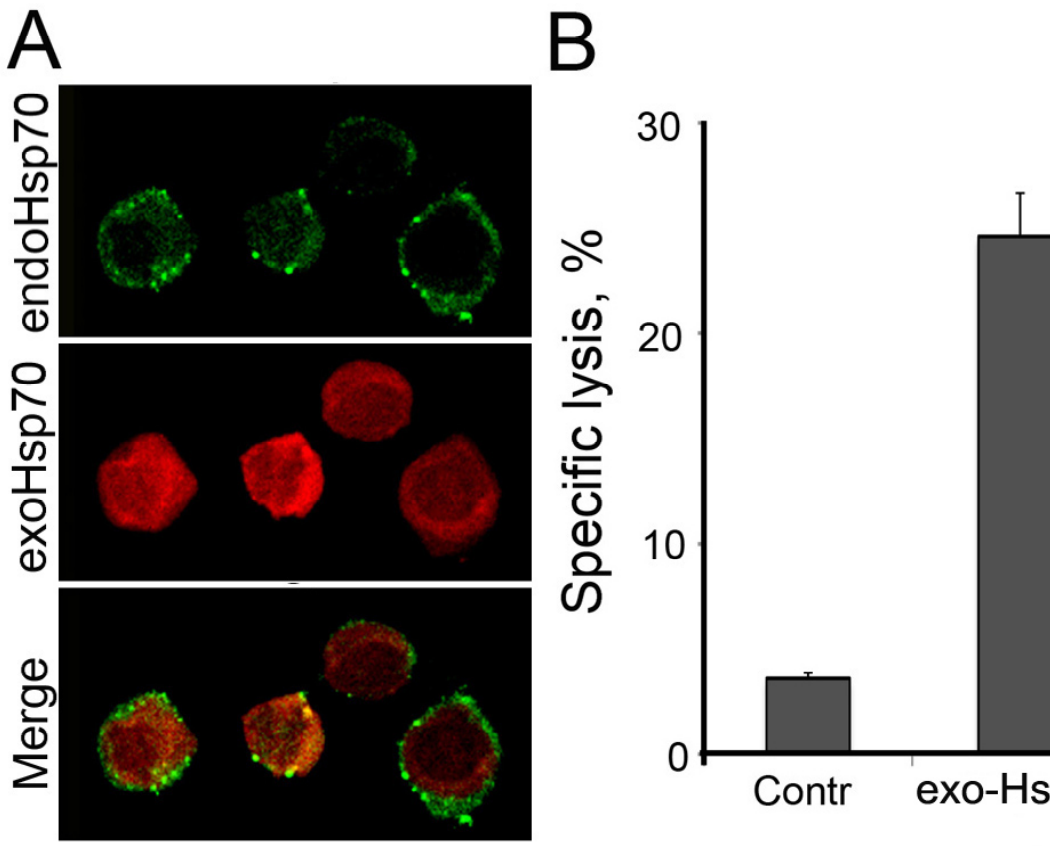

Suppl Fig. S1.The interaction of exogenous Hsp70 with human myeloid HL-60 cells leads to their sensitization to cytotoxic lymphocytes.

A. Hsp70 (20 µg/ml) labeled with Alexa555 was added to HL-60 cells and after 6 h of incubation cells were stained with SPA-810 followed by incubation with Cy2 - conjugated anti-mouse antibody.

B. HL-60 cells were incubated with 20 µg/ml Hsp70 (exoHsp70), washed and subjected to cytotoxic assay. Human peripheral blood mononuclear cells in ratio 50:1 were used as effector cells. Contr - untreated cells. The diagram represents the mean ± SD from two independent experiments.

Supplemental Figure S2

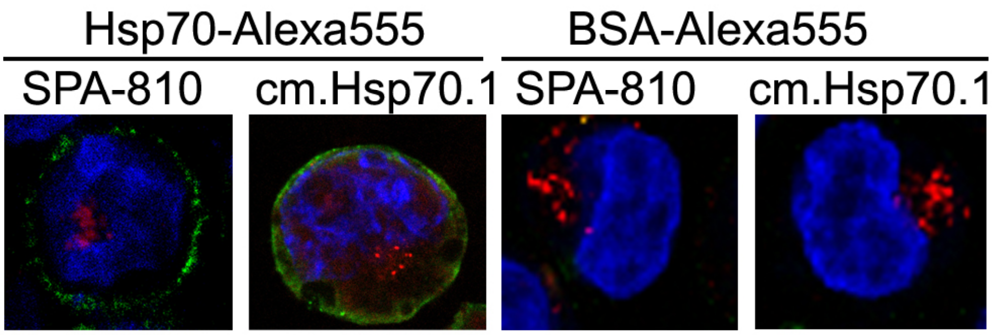

Suppl. Fig S2. Exogenous Hsp70 but not BSA expulses endogenous Hsp70 to a cell surface.

Hsp70 and BSA labeled with Alexa555 (red) were added in concentration 50 μg/ml to K-562 cell culture for 6 h. After washing cells were incubated with SPA-810 or cm.Hsp70.1-FITC antibody on ice and fixed. Cells incubated with SPA-810 antibody were stained with secondary anti-mouse antibody conjugated with Cy2 (green).

Supplemental Figure S3

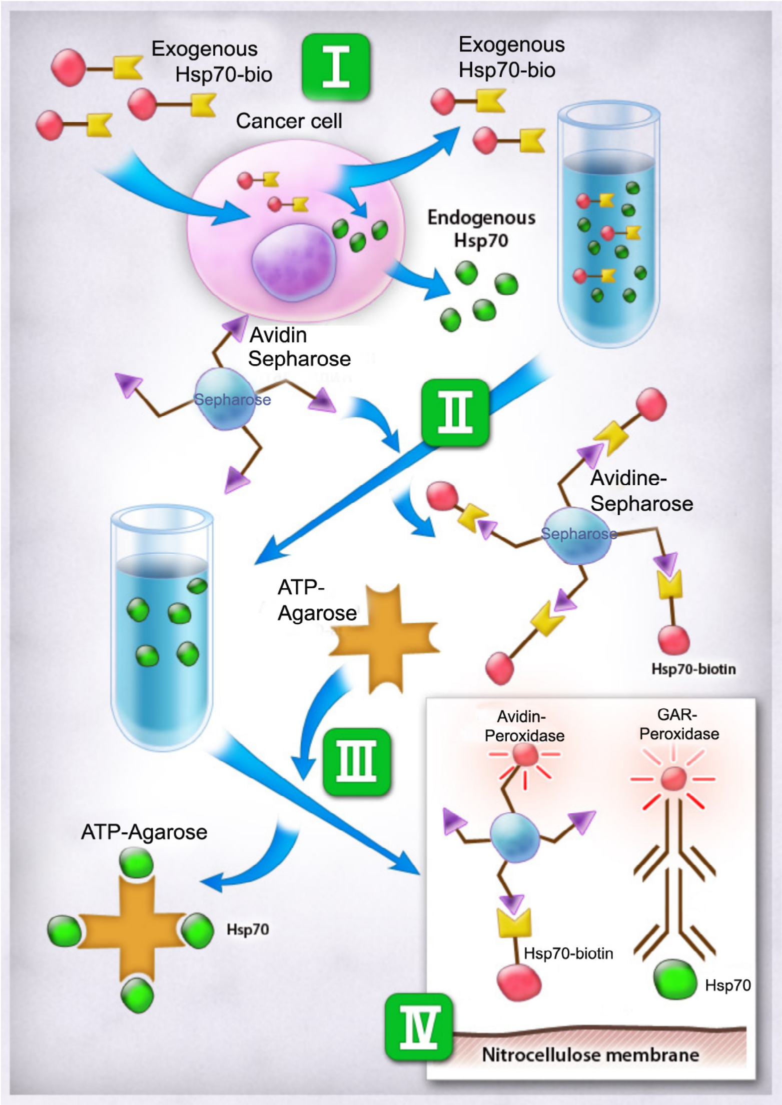

Suppl. Fig. S3. Diagram illustrated the experiment presented on Figure 7.

To discriminate between the exogenously introduced Hsp70 and the self-chaperone, C6 cells were incubated with biotinylated Hsp70 then were washed and allowed to export the endogenous proteins to the serum-free culture media. The conditioned medium was subjected to affinity precipitation first with the beads of NeutrAvidin-agarose, and the unbound material was mixed with the ATP-agarose gel. Two sets of samples, NeutrAvidin-agarose and ATP-agarose precipitates were analysed with the aid of blotting and staining with Avidin-Peroxidase or anti-Hsp70 polyclonal RSIII antibody, recognizing both the rat and human chaperones.
